# Poor Prognostic Biochemical Markers Predicting Fatalities Caused by COVID-19: A Retrospective Observational Study From a Developing Country

**DOI:** 10.7759/cureus.9575

**Published:** 2020-08-05

**Authors:** Muhammad Sohaib Asghar, Syed J Haider Kazmi, Noman A Khan, Mohammed Akram, Maira Hassan, Uzma Rasheed, Salman Ahmed Khan

**Affiliations:** 1 Internal Medicine, Dow International Medical College, Dow University Hospital, Dow University of Health Sciences, Karachi, PAK; 2 Emergency Medicine, Liaquat National Hospital, Karachi, PAK; 3 General Surgery, Liaquat National Hospital, Karachi, PAK; 4 Internal Medicine, Liaquat National Hospital, Karachi, PAK; 5 Internal Medicine, Dow International Medical College, Karachi, PAK

**Keywords:** covid-19, coronavirus, biomarkers, pandemic, mortality, infectious diseases, pakistan, severity, prognosis, survivor

## Abstract

Background and objectives

Infections with severe acute respiratory syndrome coronavirus 2 (SARS-CoV-2) are rapidly spreading, posing a serious threat to the health of people worldwide, resulting in the World Health Organization officially declaring it a pandemic. There are several biochemical markers linked with predicting the severity of coronavirus disease. This study aims to identify the most effective predictive biomarker such as C-reactive protein (CRP), ferritin, lactate dehydrogenase (LDH), procalcitonin (PCT), and D-dimer, among others, in predicting the clinical outcome of the disease.

Materials and methods

This study was conducted as a retrospective, observational, multi-centric study, including all admitted COVID-19 positive patients only. The disease outcome was followed along with the hospital course of every patient at the time of analysis. Baseline laboratory investigations of all patients were monitored both at admission and discharge. A comparative analysis was done between the survivors (n=263) and non-survivors (n=101). Statistical analysis was conducted using IBM SPSS Statistics for Windows Version 25 (Armonk, NY: IBM Corp.).

Results

Of 364 patients, 65.7% were in the isolation ward, and 34.3% were in the intensive care unit; 72.3% of patients survived, while 27.7% of patients died. The mean age of the study population was 52.6 ± 15.8 years with female patients significantly younger than male patients (p=0.001) and 50 to 75 years being the most common age group (p=0.121). Among the survivors versus non-survivors of COVID-19, there were significant differences in total leukocyte count (p<0.001), neutrophil count, (p<0.001), lymphocyte count (p<0.001), urea (p<0.001), serum bicarbonate (p=0.001), CRP levels (p<0.001), LDH (p=0.013), and D-dimer (p<0.001) at admission. At discharge, the laboratory values of non-surviving patients showed significant leukocytosis (p<0.001), neutrophilia (p<0.001), lymphocytopenia (p<0.001), decreased monocytes (p<0.001), elevated urea and creatinine (p<0.001), hypernatremia (p<0.001), decreased serum bicarbonate levels (p<0.001), elevated CRP level (p=0.040), LDH (p<0.001), ferritin (p=0.001), and D-dimer (p<0.001).

Among the recovered patients, the laboratory investigations at admission were significantly different from those at discharge like increased platelets (p=0.007), lower neutrophil count (p=0.001), higher lymphocyte count (p=0.005), an improved creatinine (p=0.020), higher sodium (p=0.008), increased bicarbonate levels (p<0.001), decreased CRP levels (p<0.001), and a lower LDH (p=0.039). However, the laboratory values of non-surviving patients had shown a lower hemoglobin (p=0.016), increased mean cell volume (p<0.001), significantly increased total leukocyte count (p<0.001), increased urea and creatinine (p<0.001), hypernatremia (p<0.001), increased bicarbonate (p=0.025), elevated D-dimer levels (p=0.043), and elevated PCT (p=0.021) on discharge. Receiver operating characteristic analysis concluded LDH (area under the curve [AUC]: 0.875), D-dimer (AUC: 0.803), and PCT (AUC: 0.769) were superior biomarkers to ferritin (AUC: 0.714) and CRP (AUC: 0.711) in predicting the fatality of COVID-19.

Conclusion

Inflammatory markers are a useful guide for predicting mortality, and the study results concluded that LDH, PCT, D-dimer, CRP, and ferritin were effective biomarkers.

## Introduction

In December 2019, a mysterious pneumonia-like syndrome was observed in Wuhan, China [1]. The causative virus was officially termed as the severe acute respiratory syndrome coronavirus 2 (SARS-CoV-2), and the disease it caused was named coronavirus disease (COVID-19) by The International Committee on Taxonomy of Viruses [2]. COVID-19 is primarily a respiratory disease but can cause various non-respiratory manifestations, such as gastrointestinal, neurological, renal, and cardiovascular symptoms [3]. The SARS-CoV-2 infection is rapidly spreading, posing a serious threat to the health of people worldwide, resulting in the World Health Organization officially declaring it a pandemic on March 11, 2020.

There are several biochemical markers linked with predicting the severity of coronavirus disease. Ferritin, produced in inflammatory conditions of the body (infectious, malignant, hematologic, and rheumatologic), is an important acute phase reactant. Microscopically, ferritin plays a defensive role within the body by limiting the supply of iron, due to which elevated serum ferritin levels can be seen in individuals with high pathogenic loads [4]. Ferritin not only limits the availability of iron to the pathogen but also regulates cytokine synthesis and release that are responsible for the cytokine (proinflammatory) storm [5].

C-reactive protein (CRP) is an acute-phase reactant that is synthesized by the liver in response to inflammation or infection. Unlike most acute-phase proteins that undergo large variations in plasma levels (depending on the synthesis, consumption, and catabolism rates), plasma CRP levels remain nearly constant. During acute inflammation, serum concentrations increase dramatically, making it a more accurate marker for sepsis [6]. CRP also contributes to the proinflammatory cycle by activating inflammatory cytokines in the body [7]. 

CRP and serum ferritin both play important roles in producing proinflammatory cytokines. Interestingly, the principal finding of immunopathology in COVID-19 is the cytokine storm. The virus replicates rapidly in the body’s endothelial and epithelial cells, resulting in the immune system developing significant numbers of proinflammatory cytokines and chemokines [1]. The severity of COVID-19 resides in the development of large quantities of proinflammatory cytokines that can eventually contribute to acute respiratory distress syndrome (ARDS) and multiple organ failure (MOF) [8]. Studies have shown that respiratory tract viral infections were associated with poor clinical outcomes due to the high rates of cytokines and chemokines released during the infection [9]. Severe COVID-19 cases are progressing quickly to complications, such as ARDS, sepsis, septic shock, metabolic acidosis, coagulopathy, and MOF [10,11]. A study that analyzed the clinical characteristics of deceased coronavirus patients identified sepsis, ARDS, respiratory failure, and heart failure as the most critical complications [12].

Due to its valuable role in the diagnosis and prognosis regarding sepsis, procalcitonin (PCT) is widely considered to be the most useful marker of severe systemic inflammation. Under normal circumstances, PCT is produced in the C-cells of the parathyroid gland. However, the rise in PCT seen during infectious states is believed to stem from neuroendocrine cells in the lungs and intestine. Its release is mediated by proinflammatory cytokines, such as tumor necrosis factor-alpha and interleukin-6. [13]. Studies found that PCT levels were lower in seriously ill patients with viral infections and were much higher in bacterial infections [14]. COVID-19 patients with elevated levels of PCT were associated with a five-fold greater risk of severe disease progression [15]. It may be because secondary bacterial infections are also common in COVID-19 pneumonia.

Severe sepsis patients also have associated high lactate dehydrogenase (LDH) levels. LDH is a cellular injury marker that shows the extent of damage to the tissue. Failure of LDH levels to normalize within 48 hours of the onset of sepsis is a strong predictor of patient mortality [16].

During sepsis, there is an upregulation of tissue factor resulting in a downregulation of anti-thrombin and a subsequent increase in plasma thrombin. At the same time, the production of protein C decreases, and upregulation of type 1 plasminogen activator inhibitor further inhibits fibrinolysis. Collectively, all these changes induce a hypercoagulable state. Increased coagulation and hypotension in sepsis can result in MOF, which is the most severe and life-threatening consequence of sepsis. A variety of molecules play important roles in the activation of the coagulation cascade. D-dimer is a sign of ongoing active fibrinolysis and, therefore, also of coagulation [17]. D-dimer is a measure of the coagulation cascade and assesses the severity of the host response, which led it to play an important role in the risk stratification of patients with sepsis to improve clinical management. A study showed that the higher the D-dimer levels, the greater the risk of sepsis and septic shock for the patient [18]. 

This study aims to identify the roles of these biomarkers (CRP, ferritin, LDH, PCT, and D-dimer), among others, in predicting the severity and clinical outcome during the disease. The identification of an effective and predictive biomarker would thus help in risk stratifying the patients and overall improving the clinical management of patients with COVID-19, especially in the region.

## Materials and methods

This study was conducted as a retrospective, observational, multi-centric study, including all the admitted COVID-19 positive patients only. The outcome of the disease was followed along with the hospital course of every patient at the time of analysis. Baseline laboratory investigations of all patients were monitored both at admission and discharge. The comparative analysis was done between the survivors (n=263) and non-survivors (n=101). The statistical analysis was conducted using IBM SPSS Statistics for Windows, Version 25 (Armonk, NY: IBM Corp.). All continuous variables were described as mean and standard deviation, and then compared using independent sample t-test and Mann-Whitney U test accordingly. Receiver operating characteristic (ROC) curves were used to determine the predictability of biochemical markers for the outcome of the disease. The Youden index was used as a summary measure of cut-off values for area under the curve (AUC). A p-value of <0.05 was considered statistically significant. All the highly significant values of <0.001 were rounded off as 0.001.

## Results

A total of 364 COVID-19 positive patients were included in the study with a mean age of 52.6 ± 15.8 years with female patients (mean age: 48.4 ± 16.4) significantly younger than male patients (mean age: 54.6 ± 15.2; p=0.001). The most common age group was 50-75 years, with two-thirds of patients being males (p=0.121). The majority of the patients were experiencing mild to moderate symptoms and were therefore admitted to the isolation ward (65.7%), while the remaining 34.3% were experiencing more severe disease and were admitted to the intensive care unit. The descriptive statistics of the study population are stated in Table 1.

**Table 1:** Demographic data of the study population (n=364) *Mann-Whitney U test to compute the p-value. **Indicates independent sample t-test used to compute the p-value. †Chi-square test used to compute the p-value. IQR, interquartile range; ICU, intensive care unit; SD, standard deviation.

No.	Characteristics	Total (n=364)	Survivors (n=263)	Non-Survivors (n=101)	P-value
1	Median age (IQR)	55.00 (43.00–65.00)	50.00 (39.25–60.75)	63.00 (55.00–70.00)	<0.001*
	Mean age (in years)	52.69 ± 15.88	49.33 ± 16.14	61.02 ± 11.63	<0.001**
2	Males (n=246), Median (IQR)	56.00 (45.00–67.00)	51.00 (43.00–64.00)	63.00 (57.00–70.00)	<0.001*
	Mean ± SD	54.63 ± 15.28	51.63 ± 15.78	62.50 ± 10.43	<0.001**
3	Females (n=118), Median (IQR)	52.00 (38.00–60.00)	43.00 (30.25–55.75)	60.00 (50.50–68.50)	<0.001*
	Mean ± SD	48.41 ± 16.41	43.95 ± 15.80	58.15 ± 13.38	<0.001**
4	Age groups				-
	0–25	16 (4.5%)	14 (5.3%)	2 (2.0%)	<0.001^†^
	26–50	135 (37.1%)	119 (45.2%)	16 (15.8%)	
	51–75	198 (54.3%)	120 (45.6%)	78 (77.2%)	
	>75	15 (4.2%)	10 (3.8%)	5 (4.9%)	
5	Hospital stay				-
	Isolation ward	239 (65.7%)	212 (80.6%)	27 (26.7%)	<0.001^†^
	ICU	125 (34.3%)	51 (19.4%)	74 (73.3%)	
6	Recovered patients (n=263)		Expired patients (n=101)		-
	Males: 180 (73.2%)	Females: 83 (70.3%)	Males: 66 (26.8%)	Females: 35 (29.7%)	0.572^†^
	Ward: 144 (87.8%)	Ward: 68 (90.7%)	Ward: 20 (12.2%)	Ward: 7 (9.3%)	0.517^†^
	ICU: 36 (43.9%)	ICU: 15 (34.9%)	ICU: 46 (56.1%)	ICU: 28 (65.1%)	0.330^†^

Among the survivors versus non-survivors of COVID-19, there were significant differences in the baseline laboratory investigations at admission, including mean hemoglobin (p=0.066), total leukocyte count (TLC) (p<0.001), neutrophil count, (p<0.001), lymphocyte count (p<0.001), monocyte count (p=0.073), urea (p<0.001), creatinine (p=0.030), serum bicarbonate (p=0.001), CRP levels (p<0.001), LDH (p=0.013), ferritin (p=0.066), D-dimer (p<0.001), and PCT (p=0.056). At discharge, the laboratory values of non-surviving patients showed thrombocytopenia (p=0.049), significant leukocytosis (p<0.001), neutrophilia (p<0.001), lymphocytopenia (p<0.001), decreased monocytes (p<0.001), elevated urea and creatinine (p<0.001), hypernatremia (p<0.001), increased chloride (p=0.044), decreased serum bicarbonate levels (p<0.001), elevated CRP level (p=0.040), LDH (p<0.001), ferritin (p=0.001), D-dimer (p<0.001), and PCT (p=0.113) (Table 2).

**Table 2:** Comparison of baseline laboratory investigations (means) among patients with COVID-19 (n=364) All p-values calculated by independent sample t-test (*significant values of <0.05). COVID-19, coronavirus disease 2019; MCV, mean corpuscular volume; TLC, total leukocyte count; CRP, C-reactive protein; LDH, lactate dehydrogenase; BNP, B-type natriuretic peptide; ESR, erythrocyte sedimentation rate.

No.	Laboratory investigation	Survivors	Non-survivors	P-value	Survivors	Non-survivors	P-value
		At admission			At discharge		
1	Hemoglobin	12.18 ± 2.40	11.66 ± 2.45	0.066	11.54 ± 2.30	11.20 ± 2.23	0.279
2	MCV	83.84 ± 8.08	81.57 ± 9.46	0.143	83.39 ± 13.38	84.49 ± 11.38	0.716
3	Platelets	232.53 ± 113.19	233.73 ± 103.85	0.928	270.94 ± 131.15	232.17 ± 147.94	0.049*
4	TLC	9.50 ± 5.19	14.10 ± 7.70	<0.001*	10.13 ± 4.48	17.74 ± 8.76	<0.001*
5	Neutrophil	70.77 ± 13.06	79.88 ± 12.13	<0.001*	71.26 ± 13.07	81.77 ± 10.50	<0.001*
6	Lymphocyte	22.34 ± 11.48	14.26 ± 9.49	<0.001*	21.44 ± 12.13	12.40 ± 8.21	<0.001*
7	Monocyte	5.58 ± 2.66	4.85 ± 3.73	0.073	5.72 ± 2.77	4.37 ± 1.99	<0.001*
8	Urea	42.64 ± 44.61	70.15 ± 51.83	<0.001*	51.88 ± 49.82	142.45 ± 74.94	<0.001*
9	Creatinine	1.50 ± 2.55	2.20 ± 2.75	0.030*	1.45 ± 1.67	3.38 ± 2.49	<0.001*
10	Sodium	138.29 ± 5.51	138.77 ± 7.45	0.511	139.12 ± 4.92	147.72 ± 9.48	<0.001*
11	Potassium	4.09 ± 0.75	4.08 ± 0.81	0.890	4.73 ± 9.58	5.70 ± 11.26	0.534
12	Chloride	103.81 ± 5.92	102.47 ± 7.29	0.074	101.08 ± 10.82	105.24 ± 14.79	0.044*
13	Bicarbonate	20.61 ± 3.40	18.98 ± 4.35	0.001*	22.66 ± 3.93	20.18 ± 5.03	0.001*
14	CRP	113.28 ± 108.74	198.67 ± 121.54	<0.001*	56.84 ± 84.79	182.94 ± 507.36	0.040*
15	LDH	495.62 ± 279.68	881.12 ± 1398.56	0.013*	465.69 ± 179.32	1298.92 ± 1810.82	<0.001*
16	Ferritin	1463.36 ± 4840.10	2757.42 ± 6483.39	0.066	1130.40 ± 1501.52	3462.06 ± 4280.59	0.001*
17	D-dimer	4.09 ± 8.07	11.58 ± 15.83	<0.001*	3.94 ± 6.88	14.56 ± 17.11	<0.001*
18	Procalcitonin	1.45 ± 8.48	4.66 ± 12.09	0.056	3.62 ± 14.80	14.83 ± 28.69	0.113
19	Troponin I	19.54 ± 69.82	35.65 ± 104.14	0.391	40.02 ± 74.49	57.95 ± 125.81	0.722
20	Pro-BNP	11779.76 ± 44060.92	4374.70 ± 6862.73	0.324	4427.70 ± 6746.50	4769.50 ± 5326.63	0.956
21	Albumin	2.93 ± 0.73	3.09 ± 0.62	0.486	-	-	-
22	ESR	46.50 ± 28.80	63.16 ± 50.94	0.174	-	-	-
23	Fibrinogen	446.66 ± 190.61	500.56 ± 248.21	0.579	-	-	-

Among the recovered patients, the laboratory investigations at admission were significantly different from those at discharge like decreased hemoglobin (p=0.020), increased platelets (p=0.007), lower neutrophil count (p=0.001), higher lymphocyte count (p=0.005), mildly increased monocyte count (p=0.049), an improved creatinine (p=0.020), higher sodium (p=0.008), lower chloride (p=0.038), increased bicarbonate levels (p<0.001), decreased CRP levels (p<0.001), and a lower LDH (p=0.039). The laboratory values of non-surviving patients had shown a lower hemoglobin (p=0.016), increased mean cell volume (p<0.001), significantly increased TLC (p<0.001), neutrophilia (p=0.074), thrombocytopenia (p=0.048), increased urea and creatinine (p<0.001), hypernatremia (p<0.001), increased bicarbonate (p=0.025), elevated D-dimer levels (p=0.043), and elevated PCT (p=0.021) on discharge (Table 3).

**Table 3:** Progress of laboratory investigations during hospital stay of patients with COVID-19 (n=364) P-value calculated by paired sample t-test (*significant values of <0.05). COVID-19, coronavirus disease 2019; MCV, mean corpuscular volume; TLC, total leukocyte count; CRP, C-reactive protein; LDH, lactate dehydrogenase; BNP, B-type natriuretic peptide.

No.	Laboratory investigation	Survivors (n=263)		P-value	Non-Survivors (n=101)		P-value
		At admission	At discharge		At admission	At expiry	
1	Hemoglobin	11.93 ± 2.29	11.60 ± 2.30	0.020*	11.75 ± 2.41	11.22 ± 2.22	0.016*
2	MCV	83.46 ± 9.85	83.33 ± 13.49	0.931	80.30 ± 10.26	84.49 ± 11.38	<0.001*
3	Platelets	239.66 ± 124.79	270.37 ± 129.57	0.007*	239.04 ± 99.54	233.85 ± 145.48	0.717
4	TLC	10.52 ± 6.05	10.12 ± 4.46	0.417	12.80 ± 6.09	17.83 ± 8.69	<0.001*
5	Neutrophil	74.63 ± 11.66	70.90 ± 13.40	0.001*	79.79 ± 10.94	81.87 ± 10.35	0.074
6	Lymphocyte	19.16 ± 10.62	21.79 ± 12.38	0.005*	14.25 ± 7.70	12.34 ± 8.13	0.048*
7	Monocyte	5.15 ± 2.54	5.73 ± 2.76	0.049*	4.90 ± 3.74	4.34 ± 1.97	0.184
8	Urea	52.02 ± 51.93	51.08 ± 48.95	0.781	71.64 ± 54.08	140.02 ± 74.57	<0.001*
9	Creatinine	1.83 ± 3.09	1.42 ± 1.64	0.020*	2.21 ± 2.84	3.33 ± 2.46	<0.001*
10	Sodium	137.64 ± 6.80	139.15 ± 4.82	0.008*	138.05 ± 8.21	147.78 ± 9.29	<0.001*
11	Potassium	4.22 ± 0.94	4.69 ± 9.37	0.594	4.10 ± 0.84	5.64 ± 11.03	0.236
12	Chloride	103.24 ± 7.15	101.09 ± 10.61	0.038*	103.61 ± 11.73	105.14 ± 14.51	0.446
13	Bicarbonate	20.03 ± 3.66	22.70 ± 4.17	<0.001*	18.80 ± 3.99	20.25 ± 4.98	0.025*
14	CRP	130.19 ± 105.48	56.84 ± 84.79	<0.001*	201.75 ± 121.21	182.94 ± 507.36	0.751
15	LDH	499.20 ± 188.40	456.69 ± 179.32	0.039*	841.47 ± 1618.16	1298.92 ± 1810.82	0.137
16	Ferritin	1409.19 ± 2012.51	1130.40 ± 1501.52	0.160	2192.10 ± 6300.94	3462.06 ± 4820.59	0.169
17	D-dimer	4.68 ± 9.47	3.94 ± 6.88	0.456	9.08 ± 13.54	14.56 ± 17.11	0.043*
18	Procalcitonin	4.44 ± 17.30	3.62 ± 14.80	0.178	3.41 ± 11.32	14.83 ± 28.69	0.021*
19	Troponin I	48.48 ± 131.09	40.02 ± 74.49	0.780	80.17 ± 173.76	57.95 ± 125.81	0.420
20	Pro-BNP	8457.10 ± 10539.14	6449.05 ± 8155.69	0.445	4234.50 ± 5055.10	4769.50 ± 5326.63	0.219
21	Fibrinogen	-	-	-	421.00 ± 516.18	476.50 ± 574.87	0.409

**Figure 1:**
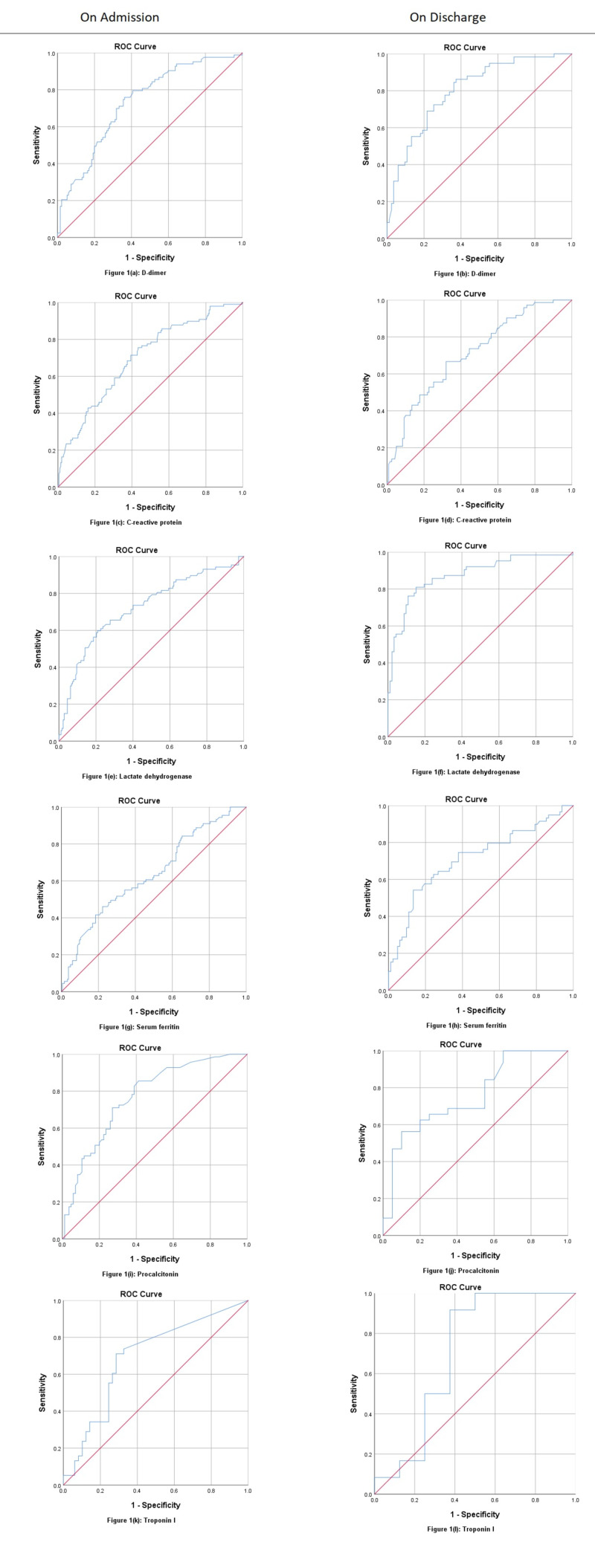
ROC curves for admitting and discharging labs of COVID-19 patients (D-dimer, CRP, LDH, ferritin, procalcitonin, and troponin I). ROC, receiver operating characteristic; COVID-19, coronavirus disease 2019; CRP, C-reactive protein; LDH, lactate dehydrogenase.

**Figure 2:**
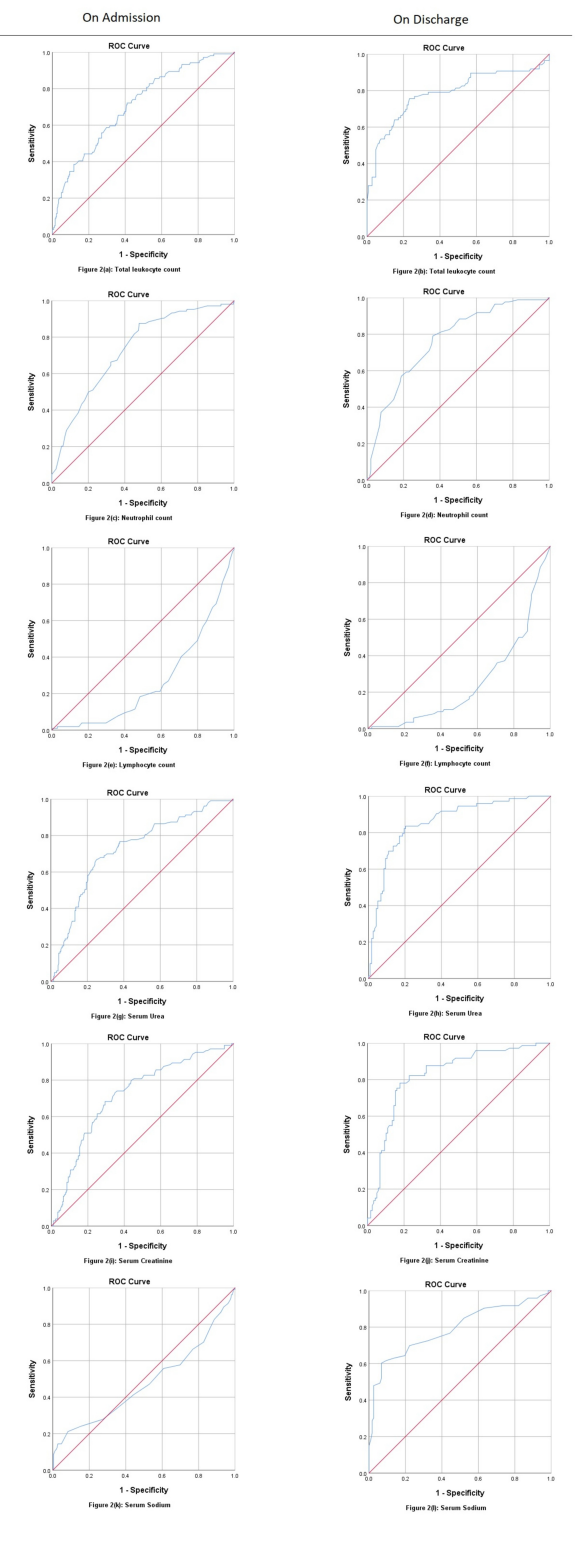
ROC curves for admitting and discharging labs of COVID-19 patients (TLC, neutrophils, lymphocytes, urea, creatinine, and sodium). ROC, receiver operating characteristic; COVID-19, coronavirus disease 2019; TLC, total leukocyte count.

ROC analysis of admitting laboratory investigations for fatalities due to COVID-19 showed PCT at a cut-off value 0.12 ng/ml (AUC: 0.769, p<0.001), predicting death with a sensitivity of 85.5% and positive predictive value (PPV) of 83.3%. D-dimer was shown at a cut-off value of 1.71 mcg/ml (AUC: 0.828, p<0.001), predicting death at 79.5% sensitivity and 82.5% PPV. Neutrophil counts were at a cut-off value of 72.50%, predicting death at 87.5% sensitivity and 91.3% PPV (AUC: 0.733, p<0.001). LDH at a 629.50 U/L cut-off value (AUC: 0.723, p<0.001) predicted death with 59.8% sensitivity and 80.0% PPV, while urea (AUC: 0.726, p<0.001) at a cut-off value of 33.27 mg/dl and creatinine (AUC: 0.719, p<0.001) at a cut-off of 1.11 mg/dl have been shown predicting death at admission. However, CRP at a cut-off of 108.30 mg/dl (AUC: 0.701, p<0.001) and ferritin at a cut-off of 1658.0 ng/ml (AUC: 0.636, p<0.001) has not been showing death predictability with good sensitivity as compared to the above parameters. ROC curves for non-surviving patients at discharge showed LDH at a cut-off value of 611.0 U/L (AUC: 0.875, p<0.001) is predicting death at 81.0% sensitivity and 86.7% PPV. Urea at a cut-off of 60.92 mg/dl (AUC: 0.860, p<0.001) has shown predicting death with a sensitivity of 83.6% and PPV of 88.7%. Creatinine at a cut-off of 1.35 mg/dl (AUC: 0.827, p<0.001) predicted death at 78.1% sensitivity and 85.8% PPV. D-dimer at a cut-off of 1.89 mcg/ml (AUC: 0.803, p=0.848) predicted death at 86.2% and PPV 86.7%. Sodium at a cut-off of 145.50 mEq/L (AUC: 0.791, p<0.001), TLC at a cut-off of 12.90 x 10^9^/L, neutrophil at a cut-off of 76.50% (AUC: 0.765, p<0.001), and PCT at a cut-off of 0.51 ng/dl (AUC: 0.755, p=0.002) have been shown predicting fatality, while ferritin at a cut-off of 909 ng/ml (AUC: 0.714, p<0.001) and CRP at a cut-off of 35.60 mg/dl (AUC: 0.711, p<0.001) again fall behind the above markers in predicting death at discharge (Table 4; Figures 1, 2).

**Table 4:** Receiver operating characteristic statistics for poor prognostic markers of COVID-19 fatalities (all-cause deaths) COVID-19, coronavirus disease 2019; PPV, positive predictive value; NPV, negative predictive value; CRP, C-reactive protein; ICU, intensive care unit; AUC, area under the curve; CI, confidence interval; SE, standard error; ROC, receiver operating characteristic; LDH, lactate dehydrogenase.

No.	Variable state	AUC	SE	95% CI	Sensitivity	Specificity	PPV	NPV	P-value
1	D-dimer (mcg/ml)								
	Admission (cut-off: 1.71)	0.733	0.034	0.666–0.800	79.5%	59.3%	82.5%	54.5%	<0.001
	Discharge (cut-off: 1.89)	0.803	0.037	0.730–0.875	86.2%	62.7%	86.7%	61.7%	<0.001
2	CRP (mg/dl)								
	Admission (cut-off: 108.30)	0.701	0.032	0.639–0.763	75.5%	56.7%	82.7%	45.7%	<0.001
	Discharge (cut-off: 35.60)	0.711	0.038	0.637–0.785	66.7%	68.1%	77.1%	55.8%	<0.001
3	LDH (U/L)								
	Admission (cut-off: 629.50)	0.723	0.035	0.655–0.791	59.8%	79.1%	80.0%	58.4%	<0.001
	Discharge (cut-off: 611.00)	0.875	0.030	0.817–0.934	81.0%	84.8%	86.7%	78.5%	<0.001
4	Ferritin (ng/ml)								
	Admission (cut-off: 1658.00)	0.636	0.036	0.565–0.706	41.6%	81.5%	74.3%	52.1%	<0.001
	Discharge (cut-off: 909.00)	0.714	0.045	0.625–0.803	74.6%	62.2%	77.3%	58.7%	<0.001
5	Procalcitonin (ng/ml)								
	Admission (cut-off: 0.12)	0.769	0.038	0.695–0.842	85.5%	58.8%	83.3%	62.8%	<0.001
	Discharge (cut-off: 0.51)	0.755	0.069	0.621–0.890	62.5%	80.0%	57.1%	83.3%	0.002
6	Troponin I (pg/ml)								
	Admission (cut-off: 0.13)	0.692	0.058	0.579–0.806	71.1%	71.4%	76.1%	65.9%	0.002
	Discharge (cut-off: 0.31)	0.708	0.139	0.436–0.980	91.7%	62.5%	83.3%	78.6%	0.123
7	Total leukocyte count (x10^9^/L)								
	Admission (cut-off: 9.31)	0.709	0.029	0.652–0.767	72.1%	58.9%	84.2%	41.0%	<0.001
	Discharge (cut-off: 12.90)	0.786	0.035	0.718–0.853	75.6%	76.7%	84.2%	65.7%	<0.001
8	Neutrophil count (%)								
	Admission (cut-off: 72.50)	0.733	0.028	0.678–0.788	87.5%	52.1%	91.3%	41.9%	<0.001
	Discharge (cut-off: 76.50)	0.765	0.032	0.703–0.827	79.1%	63.9%	83.6%	56.7%	<0.001
9	Lymphocyte count (%)								
	Admission (cut-off: 15.50)	0.267	0.028	0.213–0.322	40.4%	28.9%	55.1%	18.3%	<0.001
	Discharge (cut-off: 14.50)	0.251	0.033	0.187–0.315	36.0%	29.2%	43.3%	23.3%	<0.001
10	Urea (mg/dl)								
	Admission (cut-off: 33.27)	0.726	0.030	0.668–0.784	76.7%	62.3%	87.0%	44.9%	<0.001
	Discharge (cut-off: 60.92)	0.860	0.028	0.805–0.915	83.6%	79.7%	88.7%	71.8%	<0.001
11	Creatinine (mg/dl)								
	Admission (cut-off: 1.11)	0.719	0.029	0.662–0.777	68.3%	70.5%	84.7%	48.3%	<0.001
	Discharge (cut-off: 1.35)	0.827	0.031	0.766–0.888	78.1%	82.2%	85.8%	73.1%	<0.001
12	Sodium (mEq/L)								
	Admission (cut-off: 143.50)	0.478	0.037	0.407–0.550	21.2%	91.8%	74.1%	51.2%	0.522
	Discharge (cut-off: 145.50)	0.791	0.036	0.720–0.862	60.3%	93.2%	79.0%	84.6%	<0.001

## Discussion

The ongoing COVID-19 contagion has spurred researchers to explore effective disease severity predictors that can aid in combating the SARS-CoV-2 virus. The main purpose of this research was to establish biomarkers that could accurately predict the severity of coronavirus disease, thereby guiding clinicians in the risk stratification and clinical management of the patients. Our analysis showed that CRP is significantly elevated as compared to the mild course of the disease in severe cases of COVID-19 and was an important predictor of severity of the disease. This result is consistent with the findings of a review article that concluded that CRP amounted to 60.7% of patients infected with SARS-CoV-2 and was a crucial marker for predicting COVID-19 prognosis and mortality in these patients [19]. Qin et al. also found a significant association in their study of increased CRP and severe prognosis of the disease, but the values reported in the severe group of patients are about 2.5 times higher than ours; however, a ratio of 1.74 between the severe and non-severe groups was almost identical to our study, whereas the marker levels in the mild course of the disease are nearly identical to our levels in severe disease [20]. In their research on the use of CRP to predict disease prognosis, Gao et al. have reached the same conclusion, with CRP values reported being much higher than our analysis, although a ratio of 1:2 in the non-severe versus severe group was observed among the CRP levels close to our ratio of 1.75 [21]. A study undertaken in Wuhan, China, that evaluated the clinical characteristics of COVID-19 patients also found an important association of CRP with severe disease prognosis. However, the levels of CRP were much higher as compared to ours with a ratio of 1.67 between the severe and non-severe groups similar to our study [22]. Zheng et al. found that a mean value of 49.6 mg/L for CRP was significant for a severe prognosis of the disease, which was higher than our mean of 19.86 mg/L [11]. The conclusion reached by Zheng et al. identifying CRP as a significant marker for the severe manifestation of COVID-19 is similar to ours; however, the ratio reported between severe and non-severe cases in their study is much higher compared to our study. Their values of milder disease, along with another study, were similar to our findings [11,23].

Our study showed that LDH is also significantly increased in patients experiencing a severe course of the disease compared to those with mild infections, thereby demonstrating its role as the most potential biomarker in predicting COVID-19 severity. A study conducted in Changsha also observed LDH as an important biomarker for disease severity [23]. In that study, LDH were significantly elevated in patients with severe COVID-19; however, their mean values were three times lower as compared to our reported findings. Chen et al. also documented an association of elevated levels of LDH in patients with disease severity [24]. Their study’s levels of LDH were two times lower in both the severe and moderate groups of patients as compared to ours, but their research, as well as ours, concluded a substantial association between LDH levels and disease severity.

The significance of ferritin as a biomarker to monitor and predict disease severity as compared to CRP and LDH was much lower in our study. This conclusion differs from what is reported by many other studies, most of them identifying serum ferritin to be a significant marker for the prediction of disease severity [24,25]. A study in Wuhan, China, found a strong association of serum ferritin as a marker for the severe disease [20]. The levels of serum ferritin in our study are three times higher from the study conducted in Wuhan, in both surviving (mild-to-moderate) and non-surviving (severe) groups of patients. The levels of ferritin were much lower in that study compared to ours, but the association between the levels of ferritin and disease severity was closer to our study in terms of statistics, with our study reporting a p-value of 0.066 at admission compared to their p-value of 0.049 [24].

Our results also demonstrated the use of PCT to be a significant biomarker of the disease, which is dissimilar to the conclusions reached by Gao et al., where the authors did not find a significant association between PCT and disease severity [21]. On the contrary, several studies have reported a significant association in the elevated levels of PCT and disease severity [23,26]. This association seen in our study between PCT and its predictability of disease severity may be due to the higher rates of co-infection by bacteria and a high incidence of ventilator-associated pneumonia.

Studies have shown the role of D-dimer as an effective predictor for mortality of COVID-19 and thereby the severe course of the disease [12,27]. The values of D-dimer found in our study are significantly higher in both severe and non-severe patients when compared with the findings of other authors [24,28]. Most of the studies conducted to explore the clinical features and the role of biomarkers in predicting the severity of COVID-19 have found D-dimer to be an effective predictor, while some have associated it with increased mortality and ARDS [24,25]. Our study concluded D-dimer to be the second most effective biomarker after LDH in predicting the mortality alongside PCT, while CRP and ferritin were lagging behind the above-mentioned biomarkers in predicting mortality. Cardiac markers (trop I and pro-B-type natriuretic peptide) were not effective in predicting the severity or mortality in our study. Also, serum fibrinogen had no role in predicting severity in our findings dissimilar to another study [21]. ROC curve analysis of the same study showed fibrinogen (AUC: 0.695), which was much higher than our study, while CRP (AUC: 0.600) was much lower than our study [21]. ROC curve for D-dimer had similar sensitivity (86%) to predict severity at discharge in both the studies while our AUC was slightly lower than their study [21].

Regarding the baseline laboratory investigations, increased TLC, neutrophil count, urea, creatinine, sodium, and decreased lymphocyte count were all associated with disease severity and mortalities in our study, a finding similar to many previously conducted research studies in the region [29]. However, there were few limitations in our study, the major one being the confounding factor of various comorbidities that may be the cause of severe immune dysregulation in a certain group of patients. Other than that, secondary bacterial infections and MOF can also aggravate the immune dysregulation, which cannot be solely attributed to the viral agent being studied.

## Conclusions

We studied the effect of various biochemical markers in the prognosis of COVID-19 and the order of effectiveness among the markers. Our findings concluded that D-dimer, PCT, and LDH were superior to serum ferritin and CRP as an effective biomarker in predicting the fatality of COVID-19. We also could not establish significant associations of various other biomarkers in predicting the severity of coronavirus disease. Acute kidney injury and hypernatremia were also proven fatal events during the hospital course in our study. Such findings are crucial and can be used as guidelines when assessing the severity of the disease or treating patients in this region with the disease.
